# CANHEART: Is HDL cholesterol a cardiovascular specific risk factor?

**DOI:** 10.21542/gcsp.2016.34

**Published:** 2016-12-30

**Authors:** Mohamed Hassan, Peter Philip

**Affiliations:** Division of Cardiology, Aswan Heart Centre, Aswan, Egypt

## Abstract

Initial observational studies have identified high-density lipoprotein cholesterol (HDL-C) as an independent predictor of cardiovascular (CV) risk, even in patients on optimal statin therapy. However, the notion that higher HDL-C is better, has been seriously challenged by the results from several recent clinical and genetic trials. Data from the CANHEART study serve to clarify the relation between HDL-C and cause-specific mortality. Individuals with lower HDL-C levels were independently associated with higher risk of CV, cancer, and non-CV/non-cancer mortality compared with individuals in the reference ranges of HDL-C levels. Given the similarities in associations between HDL-C and CV as swell as non-CV outcomes, it is likely that HDL-C level serve as a marker of risk rather than a causal CV specific risk factor.

## Introduction

**Figure 1.**
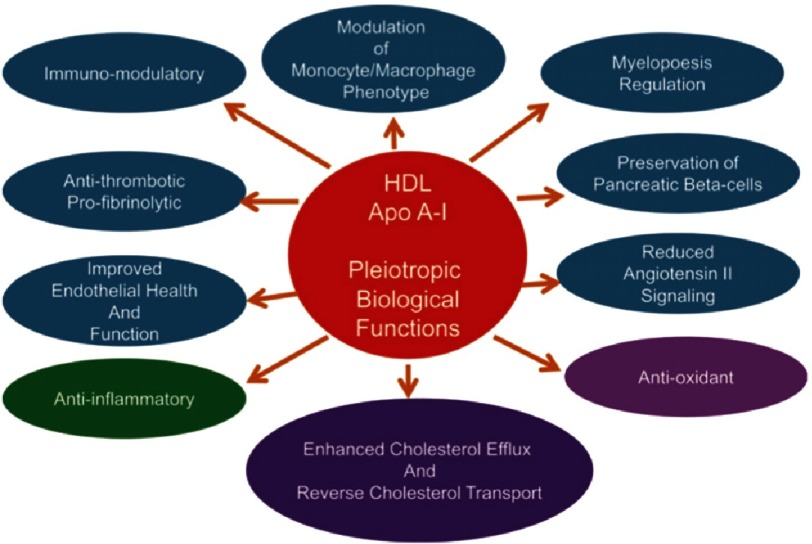
The biological functions of HDL. Adapted from [1].

HDL particles have several biological functions (Fig. 1), the most important is the ability of HDL to promotes cellular cholesterol efflux and drive the process of reverse cholesterol transport from lipid laden macrophages. Initial observational studies have identified high density lipoprotein cholesterol (HDL-C) as an independent predictor of cardiovascular (CV) risk, even in patients on optimal statin therapy^2–5^. A linear inverse relation has been reported between plasma HDL-C level and incident coronary heart disease (CHD) events, with a plateau effect at HDL-C values >90 mg/dL in men and 75 mg/dL in women^2^. A 1-mg/dl increment in plasma HDL-C level was associated with 2–3% decrement in CHD risk, and 3.7–4.7% decrement in CV mortality rates^3^.

The notion that higher HDL-C is better, has been seriously challenged by the results from several recent clinical trials^6–8^. In the Justification for the Use of Statins in Prevention: an Intervention Trial Evaluating Rosuvastatin (JUPITIR) trial cohort, HDL-C concentrations were not predictive of residual CV risk among patients treated with rosuvastatin therapy who attain very low concentrations of LDL-C^9^. Silbernagel et al. have also reported a strong association between plasma HDL-C levels and CV mortality in people without coronary artery disease (CAD), but not in patients with stable or unstable CAD^6^. In addition, higher HDL-C levels were not associated with reduced risk of vascular events in CAD patients undergoing CABG^7^. Table 1 summarizes most of the studies that examined the relation between HDL-C, CV risk, and mortality.

**Table 1:** The relationship between HDL-C, CV risk, and mortality.

Study	Number of participants	Study group	Follow-up (y)	Principle findings
Gordon et al. (1989)^3^	1,418	FHS	10.3	- A consistent linear inverse relation of HDL-C levels and CHD event rates.
	6,234	LRCF	8.5	- HDLC levels were essentially unrelated to non-CV mortality.
Four prospective American studies	1,808	CPPT	7.7	
	5,792	MRFIT	6.7	
Assmann et al. (1996)^11^	19,698	Volunteer subjects	6	A significant association between HDL-C and the incidence of atherosclerotic CHD (*P* < 0.001), which remained after adjustment for other risk factors.
PROCAM study		16–65 years		
Wilkins et al. (2014)^2^	11,515 men 12,925 women	Pooled data from 6 community- based cohorts	Men: 139,624 person-years of follow-up	A linear inverse relation has been reported between plasma HDL-C level and incident CHD events with a plateau effect at HDL-C values >90 mg/dl in men and 75 mg/dL in women.
		54–60 years	Women: 167,622 person-years of follow up	
Barter et al. (2007)^5^	9,770	NT study cohort	5	HDL-C levels were predictive of major CV events in patients treated with optimal statin therapy.
TNT trial		Patients with clinically evident CAD.		
Silbernagel et al. (2013)^6^	3,141	LURIC	8.9 ± 3.0	HDL-C was strongly associated with CV mortality in people without CHD, but not in patients with stable and unstable CHD.
	3,413	AtheroGene	4.5 ± 2.0	
	5,738	ESTHER	9.1 ± 1.6	
Angeloni et al. (2013)^7^	1,548	Patients undergoing elective CABG	2.7	Pre-operative higher HDL-C levels were not associated with reduced but rather increased MACE occurrence during follow-up.
Voight et al. (2012)^8^	20 studies	A Mendelian randomization study		- Endothelial lipase gene 396Ser allele (2.6% frequency, and associated with high plasma HDL-C levels) was not associated with risk of MI.
	20,913 MI cases			
	95,407 controls			- 1 SD increase in HDL-C due to genetic score was not associated with risk of MI.
				- However, 1 SD increase in LDL-C due to genetic score was associated with increased risk of MI.
Rohatgi et al. (2014)^12^	2,924	Dallas Heart Study	9.4	- Baseline HDL-C level was not associated with CV events in an adjusted analysis.
		(adults free from CV disease)		
				- Individuals in the highest quartile of cholesterol efflux capacity are associated with a 67% reduction in CV risk compared to those in the lowest quartile.
Ridker et al. (2010)^9^	17,802	JUPITIR trial		HDL-C concentrations are not predictive of residual vascular risk among patients treated with potent statin therapy who attain very low concentrations of LDL-C.

**Notes.**

FHS Framingham Heart Study LRCF Lipid Research Clinics Prevalence Mortality Follow-up Study CPPT Coronary Primary Prevention Trial MRFIT Multiple Risk Factor Intervention Trial PROCAM Prospective Cardiovascular Münster study TNT Treating to New Targets study LURIC LUdwigshafen RIsk and Cardiovascular health study ESTHER Epidemiologische Studie zu Chancen der Verhütung, Früherkennung und optimierten Therapie chronischer Erkrankungen in der älteren Bevölkerung (German) MACE major adverse cardiovascular events MI myocardial infarction JUPITIR Justification for the Use of Statins in Prevention: an Intervention Trial Evaluating Rosuvastatin

Recent genetic Mendelian randomization studies have also questioned the causality of inverse relationship between HDL-C and CHD risk while reaffirming the relationship of LDL-C levels and CHD risk^8^. Carriers of the endothelial lipase gene (*LIPG)* 396Ser allele (2.6% frequency, and have high plasma HDL-C levels) were expected to decrease the risk of myocardial infarction (MI) by 13% (odds ratio [OR] = 0.87, 95% confidence interval (CI) = 0.84–0.91), however no decrease in the risk of MI was detected in 396Ser allele carriers (OR = 0.99, 95% CI 0.88–1.11, *p* = 0.85)^8^. Furthermore, a 1 standard deviation (SD) increase in HDL-C due to genetic score was not associated with significant decrease in the risk of MI (OR = 0.93, 95% CI = 0.68–1.26, *p* = 0.63), which is discordant with that estimated from observational epidemiology (OR 0.62, 95% CI 0.58–0.66)^8^. In contrast, a 1 SD increase in LDL-C due to genetic score was associated with significant decrease in the risk of MI (OR =2.13, 95% CI = 1.69–2.69, *p* = 2 × 10^−10^)^8^, which is concordant with that estimated from observational epidemiology (OR =1.54, 95% CI = 1.45–1.63)^10^.

The string of failures for HDL therapies confirms the results obtained from observational and genetic studies. All existing HDL-C boosting drugs by inhibiting cholesteryl ester transfer protein (CETP), or by using extended release niacin consistently failed to have an impact on clinical outcomes in several large randomized clinical trials^13–16^. Data from the CANHEART study serves to clarify the relation between HDL-C and cause specific mortality.

## CANHEART study

The Cardiovascular Health in Ambulatory Care Research Team (CANHEART) study was an observational cohort study that was conducted by merging 17 different individual-level data sources. The essential data sources for this study included: (1) the Ontario Registered Persons Database, a registry of all Ontario residents with health insurance coverage; (2) the Canadian Institute for Health Information Discharge Abstract Database, the Ontario Diabetes Database, the Ontario Hypertension Database, and the Ontario Cancer Registry; (3) the Ontario Drug Benefit prescription database, which was used to determine outpatient prescription drug use for patients 65 years or older; (4) the Gamma-Dynacare Medical Laboratory data- base; (5) the Registrar General of Ontario Vital Statistics Database, which was used to determine cause of death of all Ontarians; and (6) the Canadian Community Health Survey (CCHS). People living in Ontario, Canada were included if they were aged 40–105 years old on January 1, 2008, without previous CV conditions or severe comorbidities, and had an outpatient fasting cholesterol measurement in the year prior to the inception date.

The study has been published recently in the *Journal of the American College of Cardiology* in November 2016^17^. The primary outcome for the study was cause-specific mortality. The cause of death was identified to be CV, cancer, or non-CV/non-cancer. A total of 631,762 individuals with a mean age of 57.2 years were included. The all-cause mortality rate was 8.1 per 1,000 person-years for men, and 6.6 per 1,000 person-years for women during a mean follow up of 4.9 ± 0.4 years. Individuals with lower HDL-C levels were more likely to have low incomes, unhealthy lifestyle, and higher triglycerides levels.

Individuals at the lowest 2 strata of HDL-C levels (≤30 mg/dl, and 31 to 40 mg/dl) had significantly higher overall mortality rates (men: 14.7 per 1,000 person-years, and 9.3 per 1,000- person years respectively; women;19.1 per 1,000 person-years, and 9.0 per 1,000- person years respectively) compared with individuals in the reference ranges of HDL-C levels. Furthermore, men at the highest HDL-C stratum (>90 mg/dl) had a higher than average mortality rate at 12.1 per 1,000-person years, while women at the highest HDL-C stratum had higher non- CV/non-cancer mortality.

Individuals with HDL-C level ≤30 mg/dl had significantly higher adjusted hazard ratio (HR) for CV mortality (men: HR = 1.81; 95% confidence interval [CI]: 1.45 to 2.25; women: HR = 2.26; 95% CI = 1.56–3.29), cancer mortality (men: HR = 1.61, 95% CI = 1.32–1.97; women: HR = 1.96, 95% CI = 1.43–2.69), and non-CV/non-cancer mortality (men: HR = 2.01; 95% CI = 1.63–2.47; women: HR: 2.86; 95% CI = 2.17–3.76) as compared to those with HDL-C of 41–50 mg/dl.

**Figure 2.**
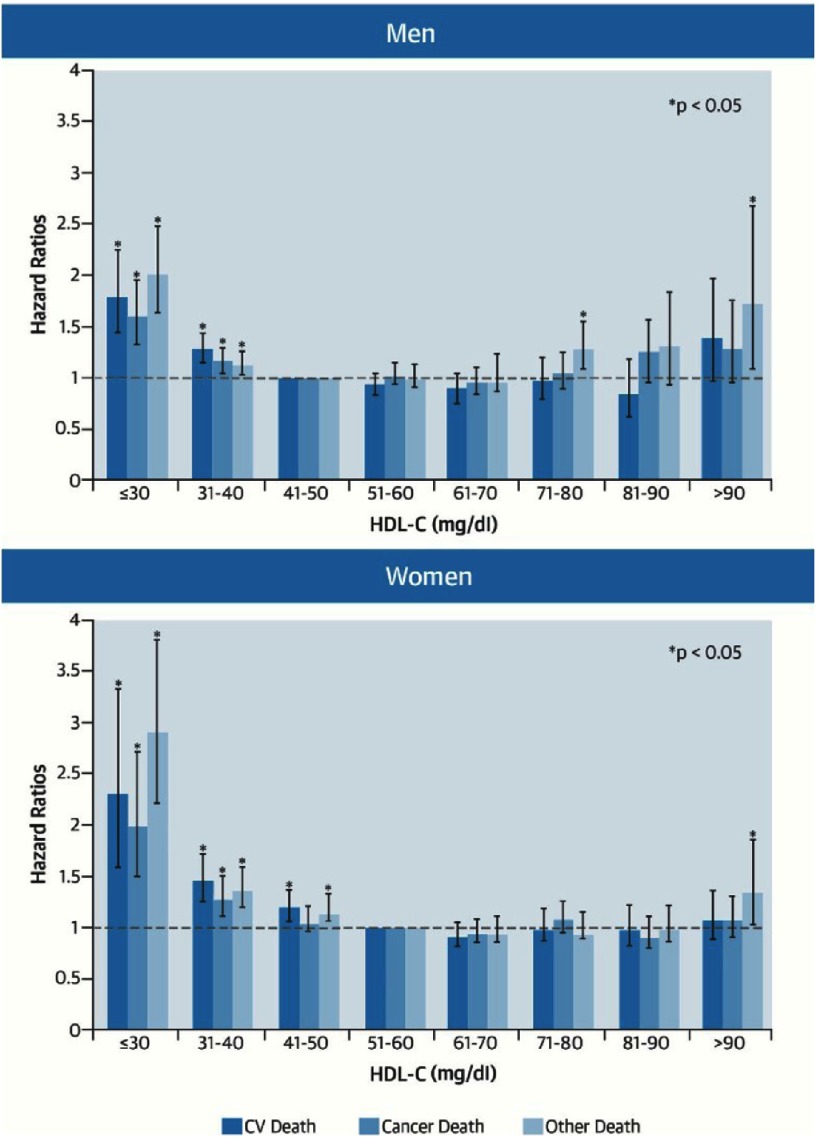
HDL-C and cause-specific mortality in individuals without prior CV disease. Adapted from [17].

On the other hand, Individuals with high HDL-C levels (>90 mg/dl) had also significantly higher HR for non-CV/non-cancer mortality. (men: HR = 1.6, 95% CI = 1.1–2.7; women: HR = 1.32, 95% CI = 1.01–1.71) (Fig. 2). Similar results were obtained for individuals who were prescribed statin therapy prior to cohort inception, or those with LDL-C level <100 mg/dl.

## Discussion

HDL particles have several protective anti-atherosclerotic properties, including the ability to mediate macrophage cholesterol efflux, antioxidant and anti-inflammatory properties, and nitric oxide promoting activity^1^. However, It is unclear whether HDL-C concentration plays a causal role in protecting against atherosclerosis. CAD may modulate the association of HDL- C with CV mortality^6^. It seems that once LDL-C is well controlled, HDL-C may be less relevant for risk assessment and risk mitigation. These findings are in keeping with the analysis of the JUPITIR trial^9^, but contradict findings from the TNT trial^5^. This could also explain the negative results of raising HDL-C with niacin or dalcetrapib in patients pre-treated with high intensity statin therapy^13–16^.

In contrast to the results of four prospective American studies that showed no significant effect of HDL-C level on non-CV mortality^3^, the CANHEART study demonstrated higher non-CV mortality in Individuals with HDL-C level ≤30 mg/dl compared to those with higher HDL-C levels. Given the similarities in associations between HDL-C and CV as well as non-CV outcomes, it is not likely that HDL-C level represents a CV-specific risk factor.

HDL-C level may not be a reliable indicator of vascular protective function of HDL. HDL particles are very complex and heterogeneous in composition and function. Moreover, the composition and function of HDLs might have been altered in patients with established CV disease. It has been reported recently that Low HDL_3_ cholesterol, but not HDL_2_ cholesterol, is associated with increased risk of death and MI^13^. Indeed, changes in HDL-C levels may not reflect changes in the physiologic functions of HDLs^14^. High HDL-C efflux capacity “the ability of HDL to accept cholesterol from macrophages”, but not HDL-C level, has been associated with a significant reduction in CV risk^12^. Furthermore, measuring HDL particle numbers and small pre-beta HDL (lipid-poor particles considered to be the major acceptors of free cholesterol from macrophages has been suggested to be a better indicator of the association between HDL and CHD risk^18^.

**Figure 3.**
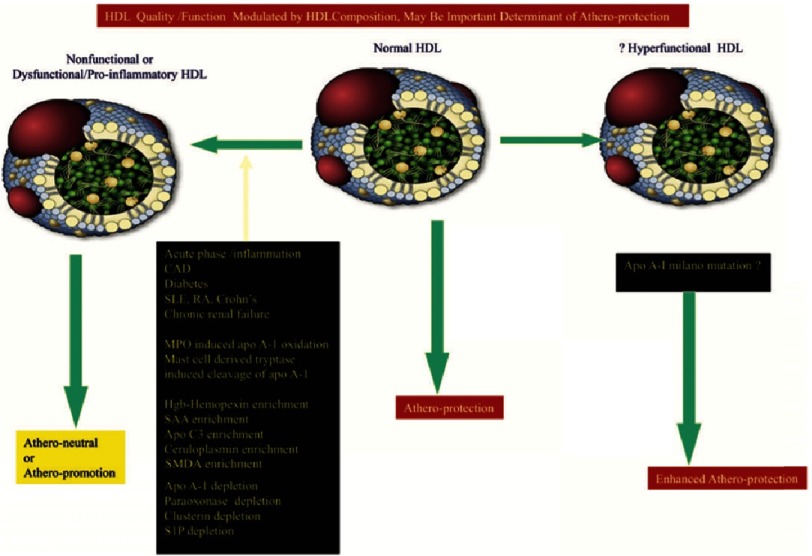
Changes in the composition of HDL in various conditions could result in non-functional or dysfunctional HDL which loses vascular protective effects or create a superfunctional HDL containing a mutant of Apo A-1. CAD, coronary artery disease; Hgb, haemo- globin; MPO, myeloperoxidase; RA, rheumatoid arthritis; SAA, serum amyloid A; SLE, systemic lupus erythematosus; SMDA, symmetric dimethy- larginine; S1P, sphingosine 1 phosphate. Adapted from [1].

Acute and chronic inflammatory states may render HDL depleted of atheroprotective molecules such as Apo A-1, paraoxonase (PON), clusterin (Apo J), and sphingosine 1 phosphate (S1P), and enriched in pro-inflammatory pro-atherogenic molecules such as serum amyloid A (SAA), haemoglobin–haemopexin complex, caeruloplasmin, symmetrical dimethylarginine (SMDA), and, importantly, Apo CIII, making the HDL particles more pro-oxidant and pro-inflammatory (so-called dysfunctional HDL) (Fig. 3)^19–21^. Indeed, the failure of the CETP torcetrapib, while often attributed to activation of the aldosterone pathway, may also have resulted from production of dysfunctional HDL^22^. The two new CETP inhibitors, evacetrapib and anacetrapib, that produce larger increases in HDL-C (>100%) and reductions (>20%) in LDL-C and do not appear to adverse have off-target effects of activating the aldosterone pathway, are currently being tested in phase III trials.

## What have we learned?

HDL-C may simply serve as a marker of risk rather than a causal CV specific risk factor. While this may be true for HDL-C level, it is clear that HDL-C is not the same as HDL. The imperative to develop bioassays that measure HDL functions in a reliable and reproducible manner is very evident.

## References

[1] Shah PK (2013). Jekyll and Hyde of HDL: A lipoprotein with a split personality. Eur Heart J.

[2] Wilkins JT, Ning H, Stone NJ, Criqui MH, Zhao L, Lloyd-jones DM (2014). Coronary heart disease risks associated with high levels of. J Am Hear Assoc.

[3] Gordon DJ, Probstfield JL, Garrison RJ (1989). High-density lipoprotein cholesterol and cardiovascular disease four prospective American studies. Circulation.

[4] Arsenault BJ, Barter P, DeMicco DA (2011). Prediction of cardiovascular events in statin-treated stable coronary patients by lipid and nonlipid biomarkers. J Am Coll Cardiol.

[5] Barter P, LaRosa J, Maroni J (2007). HDL cholesterol, very low levels of LDL cholesterol, and cardiovascular events. N Engl J Med.

[6] Silbernagel G, Appelbaum S, Scharnagl H, Silbernagel G (2013). High-density lipoprotein cholesterol, coronary artery disease, and cardiovascular mortality. Eur Heart J.

[7] Angeloni E, Paneni F, Landmesser U (2013). Lack of protective role of HDL-C in patients with coronary artery disease undergoing elective coronary artery bypass grafting. Eur Heart J.

[8] Voight BF, Peloso GM, Orho-Melander M (2012). Plasma HDL cholesterol and risk of myocardial infarction: A mendelian randomisation study. Lancet.

[9] Ridker PM, Genest J, Boekholdt SM (2010). HDL cholesterol and residual risk of first cardiovascular events after treatment with potent statin therapy: An analysis from the JUPITER trial. Lancet (London, England).

[10] Baigent C, Blackwell L, Emberson J (2010). Efficacy and safety of more intensive lowering of LDL cholesterol: A meta-analysis of data from 170,000 participants in 26 randomised trials. Lancet.

[11] Assmann G, Schulte H, von Eckardstein A, Huang Y (1996). High-density lipoprotein cholesterol as a predictor of coronary heart disease risk. The PROCAM experience and pathophysiological implications for reverse cholesterol transport. Atherosclerosis.

[12] Rohatgi A, Khera A, Berry JD (2014). HDL cholesterol efflux capacity and incident cardiovascular events. N Engl J Med.

[13] Hassan M (2014). HPS2-THRIVE, AIM-HIGH and dal-OUTCOMES: HDL-cholesterol under attack. Glob Cardiol Sci Pract.

[14] The AIM-HIGH investigators (2012). Niacin in patients with low HDL cholesterol levels receiving intensive statin therapy. N Engl J Med.

[15] Parish S, Tomson J, Wallendszus K (2014). Effects of extended-release niacin with laropiprant in high-risk patients. N Engl J Med.

[16] Schwartz G, The dal-OUTCOMES Investigators (2012). Effects of dalcetrapib in patients with a recent acute coronary syndrome. N Engl J Med.

[17] Ko DT, Alter DA, Helen Guo MK (2016). High-density lipoprotein cholesterol and cause-specific mortality in individuals without previous cardiovascular conditions. The CANHEART study. J Am Coll Cardiol.

[18] Arsenault BJ, Després J-P (2012). HDL cholesterol is not HDL–don’t judge the book by its cover. Nat Rev Cardiol.

[19] Speer T, Rohrer L, Blyszczuk P (2013). Abnormal high-density lipoprotein induces endothelial dysfunction via activation of Toll-like receptor-2. Immunity.

[20] Navab M, Reddy ST, Van Lenten BJ, Fogelman AM (2011). HDL and cardiovascular disease: Atherogenic and atheroprotective mechanisms. Nat Rev Cardiol.

[21] Riwanto M, Rohrer L, Roschitzki B (2013). Altered activation of endothelial anti- and proapoptotic pathways by high-density lipoprotein from patients with coronary artery disease: Role of high-density lipoprotein-proteome remodeling. Circulation.

[22] Barter PJ, Caulfield M, Eriksson M (2007). Effects of torcetrapib in patients at high risk for coronary events. N Engl J Med.

